# Involvement of SNX1 in regulating EGFR endocytosis in a gefitinib-resistant NSCLC cell lines

**DOI:** 10.20517/cdr.2019.15

**Published:** 2019-09-19

**Authors:** Yukio Nishimura, Kazuyuki Itoh

**Affiliations:** ^1^Division of Pharmaceutical Cell Biology, Graduate School of Pharmaceutical Sciences, Kyushu University, Fukuoka 812-8582, Japan.; ^2^Research Institute Nozaki Tokushukai (RINT), Osaka 574-0074, Japan.

**Keywords:** Lung cancer, drug-resistance, EGFR, endocytosis, endosomes/lysosomes, sorting nexin 1, membrane recycling

## Abstract

The drug gefitinib, a specific inhibitor of EGFR tyrosine kinase, has been shown to suppress the activation of EGFR signaling for survival and cell proliferation in non-small cell lung cancer cell lines. For many years, EGFR endocytosis has served as a model for investigating ligand-induced, receptor-mediated endocytosis. On EGF stimulation, EGFR is internalized and transported via clathrin-coated vesicles to early endosomes, and EGFR then recruits and phosphorylates signaling molecules, leading to the activation of downstream signaling such as MAPK/PI3K/AKT pathways-an important mechanism for regulating cell growth. Once delivered to the lysosomes, EGFR is degraded to terminate intracellular EGFR signaling via endocytosis; this process is known as receptor downregulation. Therefore, the endocytosis of EGFR is closely related with attenuation of intracellular EGFR signaling. Alternatively, EGFR is returned to cell surface from early endosomes for the continued signaling. Previous reports revealed that a competent EGF-induced endocytosis of EGFR followed by its rapid downregulation efficiently proceeds in the gefitinib-sensitive NSCLC cell lines. In contrast, gefitinib-resistant cell lines showed that EGFR endocytosis is impaired and the internalized EGFR is aggregated in the early endosomes, which is associated with the overexpressed sorting nexin 1 (SNX1), initially identified as a protein that interacts with EGFR. Thus dysregulated EGFR endocytosis is implicated in gefitinib resistance, as it leads to uncontrolled signal transduction. At present, the therapeutic relevance of EGFR endocytosis with regard to drug resistance in lung cancer has not been clarified. This review focused on the mechanism for EGFR endocytosis associated with SNX1 trafficking in gefitinib-resistant lung cancer cells.

## Introduction

The EGFR belongs to the ErbB family of receptor tyrosine kinases (RTK) and plays important roles in cell growth, survival, and cell migration. Small molecule EGFR inhibitors at targeting EGFR activity have improved the clinical outcomes of patients with many cancers^[1,2]^.

Recent studies have demonstrated that gefitinib and erlotinib - a specific inhibitor of EGFR tyrosine kinase (first-generation EGFR-TKIs) - has been shown to repress the activation of EGFR signaling required for cell survival and proliferation in the treatments of non-small cell lung cancer (NSCLC) cell lines^[3-5]^. However, many studies indicated that most patients eventually develop acquired resistance to EGFR-TKI within one year of treatment^[6,7]^: the most common mechanism is T790M in exon 20 of the EGFR gene, which is comprised of approximately 60% of EGFR-mutant patients who showed acquired resistance to first-generation or second-generation EGFR-TKI including afatinib. Afatinib was reported to show potency as compared with first-generation TKIs in inhibiting T790M kinase activity in preclinical studies^[8-11]^. However, clinical trials of afatinib revealed that most patients develop progressive diseases, possibly caused by non-selective inhibition of wild-type EGFR^[12,13]^. The third-generation EGFR-TKI, osimertinib, has also been approved for the treatment of NSCLC patients with T790M-positive NSCLC with acquired resistance to EGFR-TKIs^[14-16]^. Osimertinib is an oral, irreversible EGFR-TKI, that selectively targets both activating EGFR mutations (like L858R or exon 19 deletion) and resistant T790M EGFR mutation, as demonstrated by an in vitro EGFR recombinant enzyme assays^[14-16]^. Moreover, an acquired resistance to osimertinib has recently been reported; the mechanisms of resistance to osimertinib include L718Q or C797S EGFR mutations^[17-21]^. C797S EGFR mutation, which is located within the kinase-binding site, arises in ~33% of patients after osimertinib treatment^[22]^. Therefore, it is urgently needed to develop the fourth-generation EGFR inhibitors followed by clinical trial strategies that can conquer multiple resistance mechanisms for preventing such resistance.

Other mechanisms including alternative or bypassing pathway, leading to EGFR-independent activation, that can confer drug-resistance have been reported; MET amplification^[23]^, HER2 amplification^[24]^, PIK3CA mutation^[25]^, BRAF mutation^[26]^, and p38MAPK activation^[27,28]^. At present, no standard strategies that employ clinical agents to overcome acquired resistance to EGFR-TKI therapy after failure of EGFR TKI therapy are currently available.

Following EGF stimulation, phosphorylated EGFR (pEGFR) is endocytosed and transported to early endosomes via clathrin-coated vesicles. Then pEGFR simultaneously recruits and phosphorylates signaling molecules, activating downstream signaling pathways such as MAPK-signal transduction cascade and PI3K-AKT signaling pathway; these signaling pathways regulate cell growth and survival^[29-31]^. On the other hand, EGFR endocytosis is thought to terminate EGFR-mediated intracellular signaling, since ligand-activated EGFR is rapidly trafficked via endosomes to lysosomes. Alternatively, EGFR may be recycled back via early endosomes to plasma membrane. Previous reports suggested that dysregulation of signaling receptors including EGFR is frequently associated with cancer, since it may continue to its signaling activity from endocytic compartments and it also leads to the increased and uncontrolled receptor signaling^[32,33]^.

Previous reports showed that EGFR endocytosis is perturbed at the certain steps of vesicle trafficking from early endosomes to lysosomes in gefitinib-resistant *EGFR* wild-type NSCLC cells^[34-36]^. In contrast, EGFR endocytosis appears to be normal in gefitinib-sensitive *EGFR* mutant cells. Furthermore, intracellular SNX1 was demonstrated to be localized in the aggregates of early endosomes in which endocytosed pEGFR had also co-localized^[35,36]^. Consequently, it was inferred that membrane trafficking of EGFR may be significantly perturbed in gefitinib-resistant cells, and that the trafficking machinery of SNX1 may play a suppressive function in regulating EGFR endocytosis in human lung cancer cells.

SNX1 protein, as a constituent of the retromer, is known to play an pivotal function in regulating membrane trafficking of EGFR^[37]^, and was previously reported to interact with EGFR^[38,39]^. Furthermore, PX-domain of SNX1 specifically bound to the membranes of early endosomes; point mutations in the PX domains that cancel phosphorylated phosphatidylinositol recognition abrogate endosomes localization^[39]^. This suggests that PX-domain of SNX1 may be required for localization of early endosomes. In contrast, PX-domain mutant of SNX1 was shown to have no effect on EGFR degradation, as reported elsewhere^[40]^. As a result, the details of how SNX1 regulates the endocytic trafficking of EGFR remains uncertain at present.

It was also reported that SNX1 depletion by RNA interference promotes rapid endocytosis of EGFR in gefitinib-resistant cells^[41]^. Furthermore, the effect of SNX1 depletion on EGFR endocytosis followed by the AKT signaling pathway was investigated; a rapid change of EGFR phosphorylation followed by a significant increase in AKT phosphorylation upon ligand stimulation was demonstrated. In contrast, treatment with monensin, a membrane recycling inhibitor, significantly suppressed AKT phosphorylation in the cells^[42]^. Consequently, it was speculated that EGFR recycling pathway between the early endosomes and the plasma membrane is a pivotal step in regulating downstream activation pathway of the AKT signaling, and was further inferred that SNX1 mediates a suppressive role in EGFR recycling in the cells^[42]^. This review will mostly focus on the mechanism for EGFR endocytosis regulated by SNX1 trafficking in gefitinib-resistant human lung cancer cells.

## EGF-induced EGFR endocytosis is impeded in gefitinib-resistant human lung cancer cells

It was previously reported by using gefitinib-sensitive PC9 cells that the intracellular distribution of EGFRs is mostly distributed within late endosomes/lysosomes that stain positive for LIMPII, a typical lysosomal membrane protein, as revealed by employing double-immunolabeling confocal immunofluorescence microscopy^[34,35]^. In contrast, large amounts of intracellular EGFRs are mainly distributed in the plasma membrane of gefitinib-resistant A549 and QG56 cells^[34,35]^. Accordingly, it was hypothesized that EGFR endocytosis may be perturbed in gefitinib-resistant cells.

An internalization of EGFR was also investigated by monitoring the labeled EGF taken up by each cell line. The results revealed that efficient endocytosis of EGFR proceeds in the gefitinib-sensitive PC9 cells, therefore indicating that the EGFR is normally transported to the lysosomes in which EGFR is extensively degraded. In contrast, EGFR endocytosis is fairly suppressed in gefitinib-resistant cells. It is known that activation and phosphorylation of mutated EGFR proceeds in PC9 cells (*EGFR* exon 19 deletion) without ligand stimulation. However, in our experiments, both gefitinib-sensitive PC9 and gefitinib-resistant QG56 and A549 cell were stimulated with EGF for the analysis of EGFR phosphorylation followed by endocytosis. The results confirmed that activation status obtained from gefitinib-sensitive PC9 cells treated with EGF stimulation was the same as the cells untreated with EGF. Consequently, these observations suggested that EGFR trafficking out of early endosomes toward late endosomes/lysosomes may be abrogated in gefitinib-resistant cells, whereas EGFR endocytosis appears to proceed normally in gefitinib-sensitive cells. Also, it was shown that gefitinib inhibits efficient uptake of labelled EGF in gefitinib-sensitive cells. This suggested that an inhibitory effect of gefitinib on EGFR endocytosis appears to be more effective in gefitinib-sensitive cells as compared to gefitinib-resistant cells^[34-36]^.

Moreover, the endocytosis of EGF-stimulated pEGFR was explored in human lung cancer cells. The results revealed that large amounts of pEGFR-stained vesicles co-localized with late endosomes, were seen in gefitinib sensitive cells, whereas it was not observed any overlap of pEGFR-stained vesicles with lysosomes in gefitinib-resistant cells^[35,36]^. These results further confirmed the hypothesis that EGF-stimulated pEGFR endocytosis is considerably impeded in gefitinib-resistant cells.

## SNX1 mediates a negative regulator of EGF-stimulated EGFR endocytosis in NSCLC cells

SNX1 has been reported to be a protein that mediates lysosomal targeting of the EGFR^[37,38]^. As for the role of SNX1, it was expected that SNX1 plays an important function in membrane trafficking of EGFR and in sorting EGFR to lysosomes, since SNX1 overexpression led to the enhanced rates of ligand-stimulated EGFR degradation^[38]^.

In gefitinib-resistant cells, large amounts of SNX1 appeared to be accumulated in the early endosomes^[35,36]^. In contrast, such cluster of early endosomes stained with SNX1 was not seen in gefitinib-sensitive cells. Consequently, we speculated that EGFR endocytosis may be markedly perturbed in gefitinib-resistant cells. Accordingly, it was inferred that impairment of intracellular SNX1 function may cause a delay in EGFR endocytosis, which develops gefitinib-resistance^[35,36]^.

**Figure 1:**
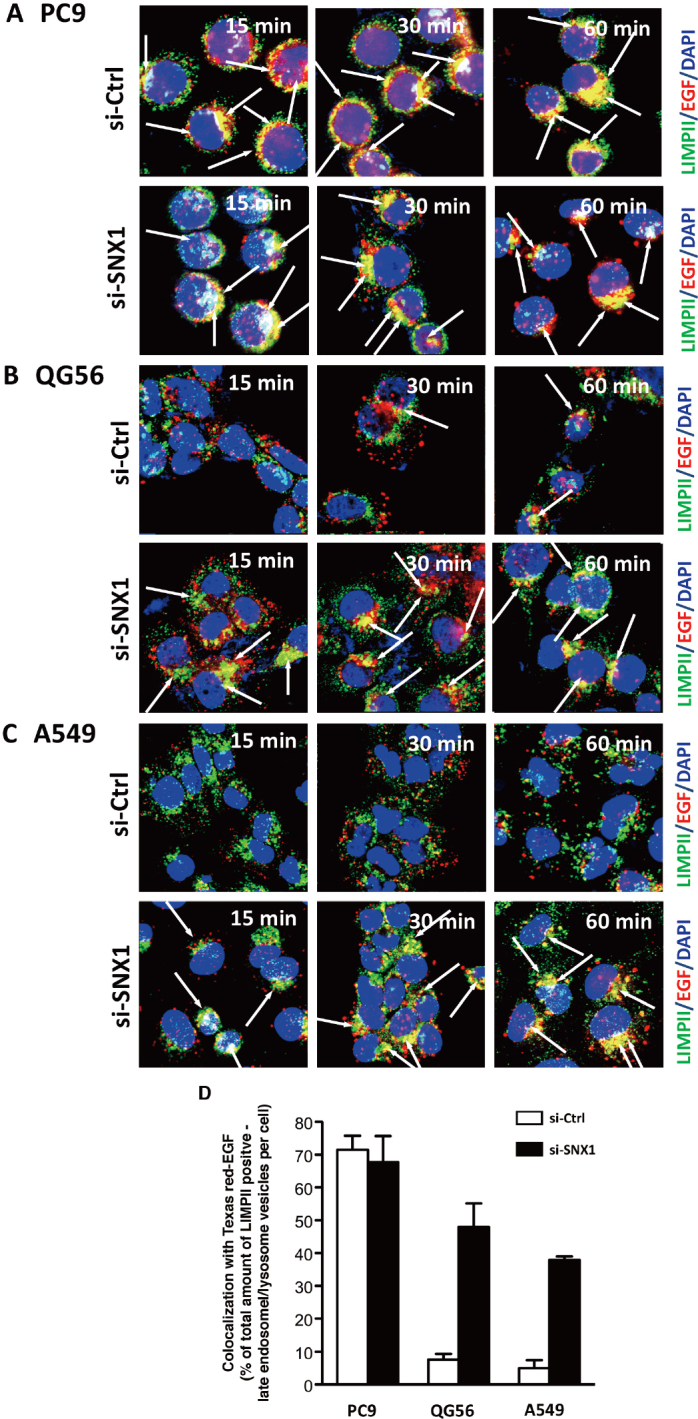
SNX1 depletion promotes efficient ligand-induced EGFR endocytosis in gefitinib-resistant NSCLC cell lines. The 3 NSCLC cell lines PC9 (A), QG56 (B) and A549 (C) cells transfected with siRNA-control (si-Ctrl) or siRNA-SNX1 (si-SNX1) were incubated with Texas red-EGF for the indicated times, and the distribution of the internalized Texas Red-EGF in the LIMPII-positive late endosomes/lysosomes was studied by confocal immunofluorescence microscopy. Superimposed images of Texas red-EGF (red) with LIMPII (green) are shown, and the merged confocal images with a yellow color are indicated by white arrows. Each cell was stained with DAPI (blue) to reveal nuclei. Quantitative analysis was carried out to determine the amounts of LIMPII-positive late endosomes/lysosomes marker that co-localized with the endocytosed Texas red-EGF following 30 min internalization in each cell transfected with si-Ctrl or si-SNX1 (D). Adapted from Nishimura *et al*.^[41]^

**Figure 2:**
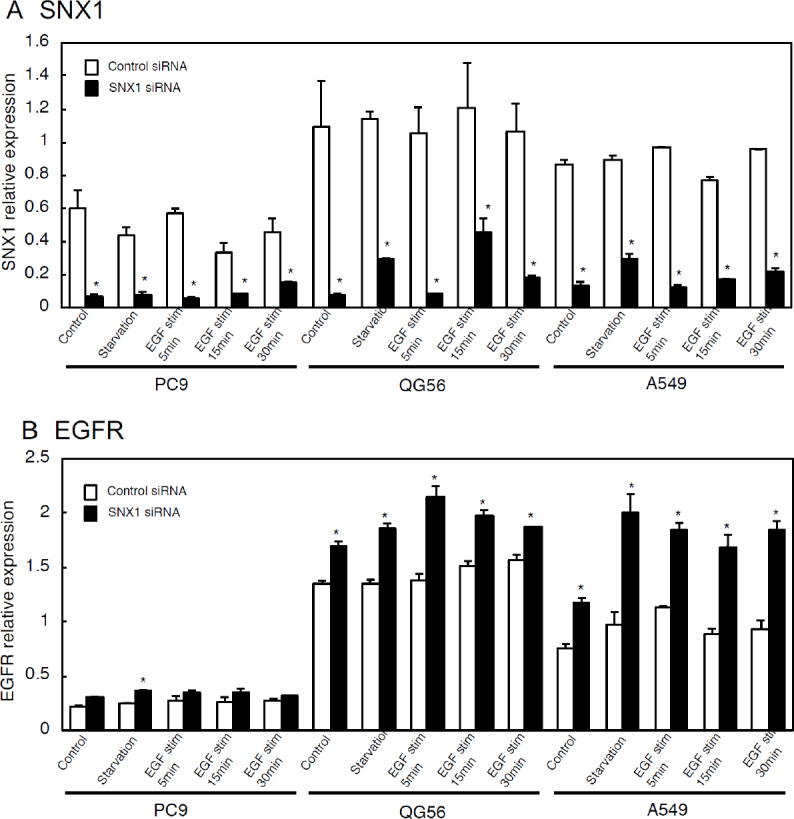
qRT-PCR analysis of SNX1 (A) and EGFR (B) mRNAs in 3 NSCLC cell lines transfected with siRNA-control or siRNA-SNX1. The 3 NSCLC cell lines PC9, QG56 and A549 cells transfected with si-Ctrl or si-SNX1 were stimulated with EGF for the indicated times, and then analyzed for the expression of SNX1 (A) and EGFR (B) mRNAs by qRT-PCR analysis. Adapted from Nishimura *et al*.^[41]^

To further explore the regulatory role of SNX1 regarding EGF-induced EGFR endocytosis, pEGFR endocytosis was analyzed by employing RNAi-mediated knockdown approaches. The results revealed that SNX1 depletion by siRNA promotes efficient endocytosis of ligand-induced EGFR in gefitinib-resistant cells as shown in Figure 1^[41]^. In contrast, the transfection of control siRNA did not affect the delayed internalization rate of the ligand-induced EGFR in the gefitinib-resistant cells. Furthermore, it was found that SNX1 mRNA is overexpressed in the gefitinib-resistant NSCLC cells; the amounts of SNX1 transcript in the gefitinib-resistant NSCLC cells is approximately 2-fold of the values, compared to the gefitinib-sensitive cells [Figure 2A]^[41]^. Additionally, it was found that SNX1 knockdown increased endogenous expression of EGFR transcript in the 3 NSCLC cell lines [Figure 2B]^[41]^. Consequently, it was postulated that SNX1 mediates a suppressive function in EGFR endocytosis in the cells. Previous report demonstrated that both the mRNA and protein expression of SNX1 were found to be decreased in colorectal cancer, compared with non-cancerous tissues, and the downregulation of SNX1 protein was closely correlated with poor differentiation and poor overall survival rate of colorectal cancer patients^[43]^. Accordingly, the detailed function of SNX1 in malignant tumor cells remains to be elucidated.

It should be stressed that SNX1 depletion induces a dramatic increase in the expression of phosphorylated form of EGFR in the cancer cell lines studied, whereas no increase of pEGFR was observed in the control siRNA-transfected cells as analyzed by western blotting^[41]^. In this regard, we infer a negative role for SNX1 on EGFR activation, thereby indicating an important function of SNX1 in the tightly regulated activation of EGFR signaling pathway.

## Monensin treatment causes a phosphorylated EGFR accumulation in early endosomes of NSCLC cells

Ligand-stimulated EGFR is endocytosed, followed by degradation in the endolysosomal network; alternatively, part of internalized EGFR is recycled back to the plasma membrane^[44,45]^. Accordingly, it was inferred that non-phosphorylated EGFR may go through considerable recycling to the plasma membrane from early endosomes.

To investigate where SNX1 controls intracellular EGFR recycling in the cells, siRNA-SNX1-transfected cells were pretreated with monensin, a membrane recycling inhibitor. After EGF stimulation, the cells were then double-labeled with antibodies to pEGFR and LIMPII or to pEGFR and endocytosed transferrin. The cellular pEGFR localization was examined by confocal immunofluorescence microscopy. The results revealed an increased pEGFR accumulation in both early and late endosomes in the monensin-treated cells^[42]^. These observations support the hypothesis that the internalized EGFR mostly returns to plasma membrane from early endosomes before being delivered to endolysosomal pathway for degradation.

Regarding the function of Rab11a; the Rab family of a small GTPase is critical regulator of intracellular vesicle trafficking and recycling^[46]^, Rab11a did not cause any pEGFR aggregation in endosomes. These results indicated that EGFR extensively returns to plasma membrane from early endosomes, but not from recycling endosomes.

## EGFR recycling upon ligand stimulation induces downstream signaling leading to the AKT activation pathway

It was expected that EGF-stimulated EGFR activation followed by downstream AKT signaling pathway may be induced by returning the internalized EGFR to plasma membrane. It is known that PDK1 phosphorylates Thr308 residue in the activation loop of AKT, resulting in the activation of AKT^[47]^, whereas PDK2 is hypothesized to phosphorylates the carboxyl-terminal site of Ser473 residue, which also leads to AKT activation^[48,49]^. Therefore, an investigation whether monensin affects AKT^S473^ or AKT^T308^ phosphorylation in SNX1 knockdown cells along with EGFR-mediated downstream signaling of AKT; control-siRNA or SNX1-siRNA-transfected cells in the absence or presence of monensin was investigated by western blot analysis^[42]^.

The result revealed that considerable increase of pAKT^S473^ was observed both in control cells and SNX1 knockdown cells. Also, the increased amounts of pAKT^T308^ were seen in these cells. On the other hand, monensin was found to inhibit the activation of pAKT^S473^ and pAKT^T308^. Furthermore, the inhibitory effect of monensin on AKT phosphorylation appeared to be forceful in SNX1 knockdown cells than in control cells. Therefore, it was speculated that monensin pretreatment significantly suppressed AKT phosphorylation, not only of pAKT^S473^ but also pAKT^T308^ both in control cells and SNX1 knockdown cells upon EGF stimulation. Collectively, it was inferred that the internalized EGFR is mostly recycled back to cell surface for downstream activation of AKT signaling pathway before being delivered to lysosomes.

## Conclusion

It was demonstrated that most EGFRs in gefitinib-sensitive cells are localized in late endosomes/lysosomes as well as in plasma membranes, indicating that EGFRs are mainly distributed in late endosomes where extensive fusion process between late endosomes and lysosomes proceeds^[34-36]^. On the other hand, most EGFRs were associated with plasma membrane of gefitinib-resistant cells. Based on these observations, we inferred that EGFR endocytosis via early/late endocytic pathway may be basically impaired in gefitinib-resistant cells, whereas EGFR endocytosis operates normally in gefitinib-sensitive cells^[34,35]^. It was previously reported that the gefitinib-sensitive PC9 cells express EGFR mutant variants, while the gefitinib-resistant QG56 cells express wild-type EGFR^[34]^. Furthermore, gefitinib-responsive lung cancers were demonstrated to express EGFR mutation with more effective to the drug than EGFR wild-type^[4,5]^. In these regards, it was hypothesized that gefitinib inhibits EGFR endocytosis in gefitinib-responsive cells more forcefully than in gefitinib-resistant cells.

Following EGF stimulation, phosphorylated EGFR is internalized by clathrin-coated vesicles, and then sorted for further transport from early endosomes, either back to plasma membrane by recycling, or to late endosomes/lysosomes for degradation: this process is known to be dependent on intracellular ESCRT pathway^[50]^. SNX1 was previously shown to interact with EGFR, and SNX1 expression stimulated ligand-dependent EGFR degradation in lysosomes, thereby indicating that SNX1 functions to enhance EGFR trafficking in the endosomes to lysosome pathway^[38,39]^. Accordingly, it was suggested an important function for SNX1 in regulating EGFR-mediated signaling. The gefitinib-resistant mechanism mediated by SNX1 remains to be uncertain at present. Consequently, it should require further studies to understand the important function of SNX1 associated with endocytosis and signaling of EGFR.

With regards to the role of SNX1 involved in EGF-induced EGFR endocytosis, associated with the downstream activation of AKT pathway, a substantial amounts of pEGFR accumulation was seen in early endosomes of gefitinib-resistant cells in the presence of monensin and SNX1 depleted with siRNA^[42]^. This was followed by suppressed AKT phosphorylation. The observed inhibition of AKT phosphorylation was much potent in SNX1-depleted cells than in control cells. Also it was shown that an increase of phosphorylated p44MAPK was noticeably suppressed by monensin treatment. Therefore, it was speculated that monensin has a stronger inhibitory effect on EGFR-mediated downstream signaling such as AKT/MAPK pathway in the absence of SNX1. These data support a model by which SNX1 plays a suppressive role in regulating EGFR trafficking to the endosomes/lysosomal degradation pathway and EGFR recycling toward plasma membrane.

It should be stressed that monensin had no effect on HGF-stimulated MET RTK activation, followed by AKT phosphorylation. This indicates that the RTK MET may not be involved in signaling from the endosomal recycling pathway in NSCLC cells^[51]^. Further analysis of MET and the recycling pathway of MET may be required to exploit future cancer therapies.

Based on this data, it was suggested that EGFR recycling to cell surface upon ligand stimulation is a pivotal step that induces downstream activation of AKT pathway. In this regard, suppressive regulation of EGFR endocytosis and EGFR recycling controlled by SNX1 may be a crucial step for the maintenance of EGFR-mediated pathway.

**Figure 3:**
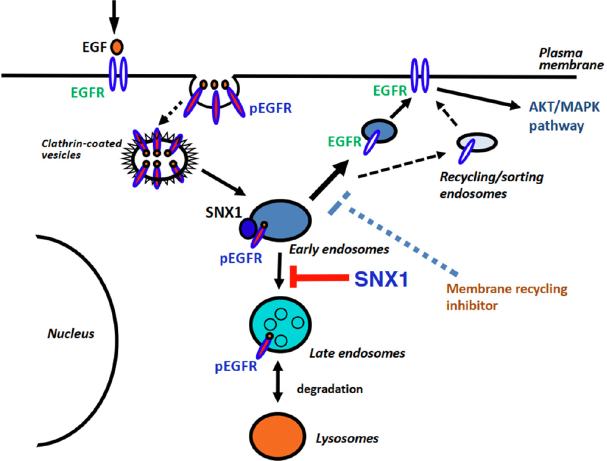
Schematic diagram for SNX1-mediated endocytic trafficking of EGFR in NSCLC cells

A proposed mechanism for the negative regulation of EGFR endocytosis and membrane recycling associated with SNX1 trafficking in human NSCLC cells is illustrated in Figure 3. It should be considered that targeting the EGFR recycling pathway in combination with EGFR-TKI therapy may be an important strategy for lung cancer.

## Declarations

### Copyright

© The Author(s) 2019.
